# Experiences with Recruitment of Marginalized Groups in a Danish Health Promotion Program: A Document Evaluation Study

**DOI:** 10.1371/journal.pone.0158079

**Published:** 2016-06-23

**Authors:** Marianne Rasmussen, Eva Kanstrup Poulsen, Anne Stoffersen Rytter, Tine Mechlenborg Kristiansen, Carsten Kronborg Bak

**Affiliations:** 1 Department of Health Science and Technology, Aalborg University, Aalborg, Denmark; 2 University College of Northern Denmark, Aalborg, Denmark; Leibniz Institute for Prevention Research and Epidemiology (BIPS), GERMANY

## Abstract

**Background:**

Studies have found that marginalized groups living in deprived neighborhoods are less likely to participate in health programs compared to the majority of society. This study evaluates recruitment approaches conducted during a national government-funded project in 12 deprived neighborhoods across Denmark between 2010 and 2014. The aim of this study was to understand how recruitment approaches could promote participation in health programs within deprived neighborhoods to reach marginalized groups.

**Method:**

Documents from all 12 of the included municipalities were collected to conduct a document evaluation. The collected documents consisted of 1,500 pages of written material with 12 project descriptions, three midterm and 10 final evaluations. The collected data were analyzed through a qualitative content analysis.

**Results:**

The results are based on the fact that only 10 municipalities have developed evaluations related to recruitment, and only three evaluations provided a description of which marginalized groups were recruited. Challenges related to recruitment consist of difficulties involving the target group, including general distrust, language barriers and a lack of ability to cope with new situations and strangers. Additional geographical challenges emerged, especially in rural areas. Positive experiences with recruitment approaches were mainly related to relationship building and trust building, especially through face-to-face contact and the project employees’ presence in the neighborhood. Additionally, adjusting some of the interventions and the recruitment strategy increased participation.

**Conclusion:**

This study found that relation and trust between the residents and the project employees is an important factor in the recruitment of marginalized groups in deprived neighborhoods as well as adjusting the health interventions or recruitment strategy to the target groups. In future research, it is necessary to examine which recruitment approaches are effective under which circumstances to increase participation among marginalized groups.

## Introduction

In recent decades, there has been an international focus on reducing social inequalities in health [1,2]; nonetheless, inequalities with regard to health appear to be increasing [2]. The problem involves individuals with poor connections to the labor market and transfer income; these individuals are often more vulnerable to poor health outcomes [3]. The Danish Health Authority has defined these individuals as marginalized groups [4]. Marginalized people often live in deprived neighborhoods. A “*deprived neighborhood*” is defined as a geographically bounded area with a high proportion of adults with low socioeconomic status, characterized by indicators such as unemployment, low income, low education and low-paid jobs [5]. Within health science disciplines, the field of research on neighborhood conditions and their impact on individuals has grown. This area has attracted substantial interest among researchers in recent years [6]. It has been demonstrated that the health of individuals varies according to neighborhood characteristics, and several studies have proven that socioeconomically poor areas have higher morbidity and mortality rates than other areas [7–11]. Furthermore, studies have shown that those who live in deprived neighborhoods are less likely to participate in health programs and in health research compared to the majority of society, which have a higher socioeconomic status. [12–16]. The challenges within deprived neighborhoods vary substantially and involve financial or transport-related barriers, distrust or fear of authorities, communication difficulties, and lack of information and awareness in relation to health programs [14,16]. A systematic review by Bonevski et al. (2014) that involved several different recruitment approaches found no clear strategy that could improve the participation rate, though the best method did appear to be involvement in the community in an attempt to reach marginalized groups. The groups of interest were described as socially, culturally or financially disadvantaged groups compared to the majority of society. Involvement in the community should be in collaboration with groups and organizations in the community and should involve community members in the design of the health interventions [16]. Another review emphasized the lack of evidence regarding which recruitment approaches for residents living in deprived neighborhoods increase participation in health programs [12].

From 2010–2014, 12 municipalities in Denmark were involved in a national government-funded project, *Forebyggelsesindsatser i nærmiljøet* (FIN) (Prevention efforts in the local community), which aimed to strengthen health and wellbeing among marginalized groups in deprived neighborhoods. The Danish Health Authority committed these municipalities to work with predetermined risk factors of mental health, smoking and physical activity as well as either diet or alcohol use. Testing of methods for recruitment of the marginalized groups was also part of the project’s framework [4].

The knowledge gained from FIN and the limited evidence and research on the recruitment of marginalized groups provides a strong argument for further research on how to recruit marginalized groups from deprived neighborhoods in an attempt to increase participation in local health-promoting interventions. Thus, the aim of this article is to understand how recruitment approaches can promote participation in health programs within deprived neighborhoods to reach marginalized groups. We investigated this issue through a document evaluation of the 12 participating municipalities’ experiences with the recruitment of marginalized groups during the 2010–2014 period.

## Method

### The empirical setting

In 2010, the Danish Health Authority invited the Danish municipalities to apply for participation in and subsidies by the FIN project. The target group was residents who lived in deprived neighborhoods. The neighborhoods were either a deprived area in a larger town, a smaller town or an area composed of two or three small villages in rural areas. A selection criterion was that the municipalities could document the need and potential for interventions in a neighborhood with many marginalized people. Additional criteria were the establishment of new preventive and health promotion interventions, the geographical spread of the municipalities, previous experiences with interdisciplinary interventions, the potential for maintenance, and municipal co-financing [4]. Of the 98 municipalities in Denmark, 45 applied, and 12 municipalities were selected based on their applications.

The types of the interventions were not specified in advance by the Danish Health Authority. Therefore, the project employees had an open framework to shape the local interventions as long as they were aimed at the predetermined risk factors: mental health, smoking, physical activity, and diet or alcohol. The local interventions ranged widely, including exercise classes, smoking cessation courses, cooking teams and other social activities. Other interventions were more creative; for example, one municipality used activities with art to promote mental health.

As part of participation in the FIN project, the municipalities were required to conduct a local cross-sectional survey in the neighborhood. The survey was designed to obtain a better understanding of the individual neighborhoods. Furthermore, the survey made it possible to identify more specific target groups among the residents in the neighborhoods and by this develop health interventions. All municipalities identified target groups that had health problems, though few of the municipalities used these data in relation to design the recruitment of the residents. Some municipalities decided to work with all residents—as an unitary target group and some municipalities prioritized the most vulnerable groups in the neighborhoods based on the findings from the survey. The vulnerable groups often scored high on parameters such as loneliness, low attachment to the labor market, and had both physical and mental health problems according to the survey.

A comparison of the 12 deprived neighborhoods included in FIN (anonymized and termed A-L) showed that they had certain common characteristics: a higher proportion of residents with a low educational level, with lower incomes and who received social assistance compared to residents in other parts of the municipalities. Diversity was also present in the 12 neighborhoods, as illustrated in Table 1. The number of households ranged from 203–3,190, the number of residents ranged from 491–6,376, the percentage of immigrants or descendants ranged from 2%-52%, and the locations included nine urban and three rural areas. This diversity illustrates the different possibilities and working conditions for the project employees in the 12 neighborhoods.

**Table 1:** Neighborhood characteristics.

Neighborhood	Location^a^	Households	Residents(total number)	Immigrants or descendants^b^ (%)	Low education level^c^ (%) (The neighborhood (the municipality in general))	Average gross income^d^ (DKK) (The neighborhood (the municipality in general))	Residents receiving social assistance^e^ (%) (The neighborhood (the municipality in general))
A	Urban	1,690	3,181	7	51.1 (39.9)	220,827 (256,376)	11.7 (9.9)
B	Urban	1,991	4,077	30	49.8 (35.5)	220,280 (265,545)	18.2 (11.4)
C	Urban	575	1,068	32	60.9 (43.3)	170,584 (245,387)	18.4 (8.0)
D	Urban	963	1,441	50	60.9 (38.3)	190,790 (270,562)	20.8 (9.9)
E	Urban	1,110	2,138	32	59.5 (36.6)	211,841 (276,087)	15.4 (9.7)
F	Rural	1,755	2,957	3	52.0 (47.7)	216,607 (223,614)	4.1 (6.9)
G	Rural	203	491	37	67.4 (47.8)	151,828 (225,545)	19.8 (9.8)
H	Urban	638	1,136	20	57.6 (35.6)	186,847 (278,406)	18.1 (8.7)
I	Rural	1,586	3,468	2	47.2 (43.4)	239,029 (252,089)	6.3 (5.9)
J	Urban	741	1,434	52	67.6 (37.1)	155,238 (245,116)	24.8 (7.1)
K	Urban	617	1,066	35	64.5 (45.8)	176,059 (246,204)	18.2 (6.0)
L	Urban	3,190	6,376	26	57.7 (35.5)	178,856 (249,318)	22.8 (9.0)

The table shows the 12 neighborhoods’ characteristics at the start of FIN [17].

^a^ A neighborhood is defined as *urban* if it was located in or close to a larger city, defined as a city with more than 3,000 citizens, and *rural* if it is located in an area in which more than half of the people lived outside cities with more than 3,000 citizens or at a distance to a larger city [18].

^b^ *Immigrants* are defined as persons born in foreign countries, of whom neither of their parents were born in Denmark and were Danish citizens. *Descendants* are defined as persons born in Denmark, of whom neither of their parents were born in Denmark and were Danish citizens [19].

^c^ Primary school as highest education level or education level unknown. The bracket shows the percentage for the municipality.

^d^ The average gross income in Denmark (2008) is 271390 DKK [20]. The bracket shows the average gross income for the municipality.

^e^ Danish citizens can receive social assistance if they are unemployed and financially unable to support themselves and their families [21]. The bracket shows the percentage for the municipality.

### Data collection of documents

The data materials in our study were collected in the spring of 2015. Contact with the 12 municipalities was made through email, and we requested documents in the form of project descriptions as well as midterm and final evaluations. Because the project description was a mandatory document, it was possible to obtain 12 project descriptions. Project evaluation was not mandatory for the municipalities, and it was only possible to collect three midterm and 10 final evaluations. Additionally, six documents from researchers or external evaluators were included. The collected documents consisted of 1,500 pages of written material. An overview of the documents and their content, including whether the municipalities described, evaluated and adjusted the recruitment approach, is shown in Table 2. The project descriptions are included in the analysis to identify recruitment approaches, and the evaluations are included to assess recruitment strategies.

**Table 2:** Overview of documents and content.

Neighborhood	A	B	C	D	E	F	G	H	I	J	K	L
Project description (2010)	✓	✓	✓	✓	✓	✓	✓	✓	✓	✓	✓	✓
Midterm evaluation (2012)	−	✓	−	−	✓	−	−	−	−	−	✓	−
Final evaluation (2014)	✓	✓	✓	✓	✓	✓	−	✓	✓	✓	✓	−
Documents or input from researchers or external evaluators included	✓	−	−	✓	✓	✓	−	−	−	✓	✓	−
Description of the recruitment approach	✓	✓	✓	✓	✓	✓	✓	✓	✓	✓	✓	✓
- Project description												
Evaluation of recruitment approach	✓	✓	✓	✓	✓	✓	−	✓	✓	✓	✓	−
- Midterm or final evaluation												
Evaluation of recruitment in relation to marginalized groups	0	0	0	0	✓	0	−	0	0	✓	✓	−
- Midterm or final evaluation												
Adjustment of recruitment approach	✓	✓	✓	✓	✓	✓	−	0	0	✓	0	−
- Midterm or final evaluation												

(✓) Document or information exists.

(−) Document does not exist.

(0) Missing information.

Ten municipalities (A, B, C, D, E, F, H, I, J, K) evaluated approaches in relation to recruitment, and only three (E, J, K) provided a description of which residents were recruited to determine whether the residents belonged to one of the marginalized groups in the neighborhood. Municipality E described a special focus on three target groups: immigrants and their descendants, residents experiencing economic hardship and the lonely, who are mentally vulnerable. These groups were estimated to constitute approximately 40% of the 1,700 residents over 18 years of age. In municipality J, the most vulnerable populations are described as men of Danish origin who had indicated that they felt lonely in the survey, residents with an ethnic background other than Danish who are outside the labor market, and residents with chronic pain and health problems. Municipality K was not as specific in the evaluation as E and J. It divided the group of residents into two general categories based on project employees’ experience: vulnerable and resourceful. Two municipalities (G, L) did not compose an evaluation. Seven municipalities (A, B, C, D, E, F, J) described adjustments to the recruitment approach in their evaluations.

### Data analysis

FIN ended in 2014. Therefore, it was possible to conduct a summative process evaluation of the municipalities’ work with recruitment to provide an understanding of why and how the municipalities' interventions did or did not work [22]. We analyzed the documents through a qualitative content analysis, defined as an interpretation of the content of text data through the systematic classification process of coding and identifying themes or patterns [23]. Authors MR, EKP and ASR conducted the content analysis. To ensure consistency and quality throughout the analysis process, all documents from one municipality (F) were coded individually and subsequently reviewed and discussed by all three authors. This approach aimed to produce a shared systematic review of the documents to ensure that nuances in the analysis of the documents were initially coded individually before being summarized in a joint process.

**Fig 1:**
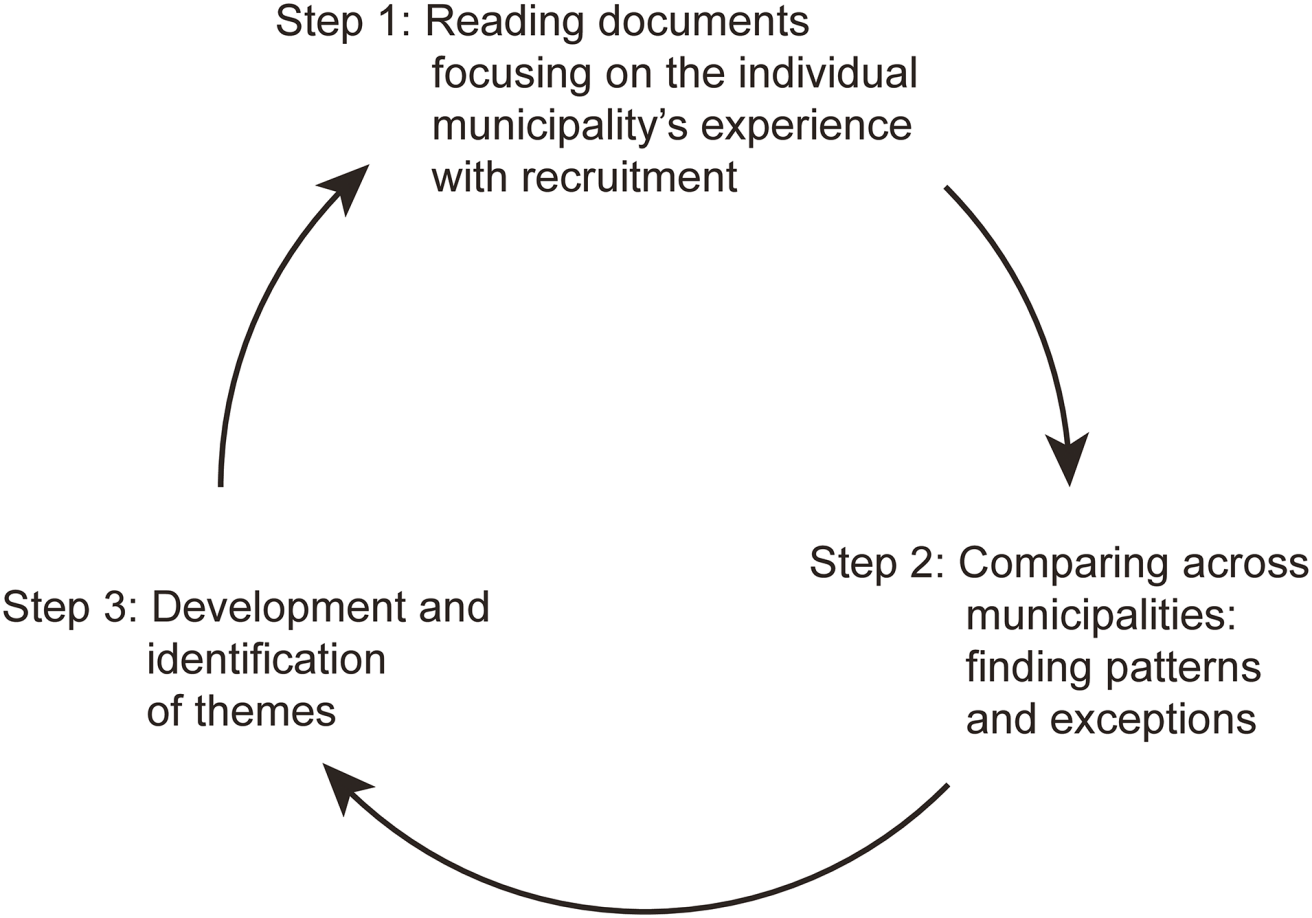
Data analysis process. Illustration of the steps in the data analysis process from reading documents to identifying themes.

A comparison of documents from the project description, the midterm and the final evaluations provide insight into the development of the municipalities’ work with recruitment from start to finish. This approach made it possible to analyze whether the purpose, ideas and interventions at the beginning of the project were reached at the end [22]. We carefully analyzed the documents, looking for patterns, themes, and regularities as well as contrasts, paradoxes, and irregularities. The “negative” exceptions as well as the “positive” patterns were highlighted [24]. By comparing and analyzing experiences across municipalities, it was possible to generalize experiences from the individual municipality. The data analysis process is shown in Fig 1.

The identification of themes or patterns was a circular process. As the themes emerged, it was sometimes necessary to return to the documents and focus on an individual municipality’s experience with recruitment. Authors CKB and TMK are experienced researchers in deprived neighborhoods and qualitative methods, respectively. MR, EKP and ASR discussed the analysis process and the identified themes with CKB and TMK throughout the entire process and linked the findings to prior knowledge in the fields of recruitment, marginalized groups and deprived neighborhoods.

## Results

Through the content analysis, we identified the recruitment approaches in the municipalities. Two main themes emerged in relation to recruitment. One theme included *challenges* with recruitment, and the other included *positive experiences*.

### Recruitment approaches

The following recruitment approaches were identified in the documents: posters, social media, personal contact (face-to-face, telephone or letter), newspaper, radio, flyers and the use of collaborators through, for example, libraries, health consultants, and nurses. Unfortunately, not all of these approaches were evaluated in the documents.

The content analysis found that the evaluation of the recruitment approaches included qualitative interviews with residents and project employees (A, B, C, D, E, F, H, I, J, K) as well as quantitative data in terms of the amount of participation (A, B, C, D, E, F, I, J, K). However, the amount of participation was unsuitable because it did not provide descriptions of the potential number of participants who could be included and whether the residents who participated were part of the targeted marginalized groups. As an example, one municipality (J) merely stated that 10 residents participated in one of the exercise groups.

### Challenges

Many municipalities experienced difficulties in reaching the target group, and several of the original ideas for interventions were cancelled due to too few participants or no attendance (A, B, C, E, F, I, J, K). The main challenges within the recruitment approach were divided into two themes: *challenges with the target group* and *geographical challenges*.

#### Challenges with the target group

Some of the challenges were related to and complicated by the project employees’ dialogue with the residents. In some documents, a general distrust of the government is described as problematic (D, E).

*"The residents who do not use Healthy Zone [project name] have a different view on the relation to the municipality*. *There is skepticism and concern that if the employees in Healthy Zone 'discover' that there are difficulties in a home*, *they may go to the municipality with the knowledge*, *or they worry that the municipality*, *if a resident on early retirement pension works as a volunteer in activities*, *will determine that one is more able to work than anticipated*, *and thus one will be afraid to be stripped of his pension*.*"* (E, final evaluation) (Our own translation—edited by American Journal Experts).

The residents’ distrust made it difficult for the project employees to initiate a conversation about the project and its interventions. Therefore, it was difficult to create motivation and participation.

Furthermore, the dialogue was complicated by language barriers related to the fact that there were many different nationalities and a high proportion of illiterate residents in several neighborhoods (B, D, E, H, I, J).

*”It seems that especially the non-ethnic Danes are difficult to recruit through written materials*, *and several of them say openly that they do not read the material that is distributed*. *Therefore*, *the experience of others and ‘word of mouth’ is an important method for recruiting this particular group of residents*.*”* (E, final evaluation) (Our own translation—edited by American Journal Experts).

Language barriers can complicate recruitment and might be a reason for the residents’ lack of awareness of the interventions because immigrants may have difficulties receiving the information. Therefore, recruitment strategies such as posters, social media, newspapers, radio or flyers might be less effective in neighborhoods where a large part of the marginalized groups are immigrants with linguistic challenges.

In addition to distrust and language barriers, the analysis indicates that the lack of participation among marginalized groups could be explained by the lack of capacity and resources to be able to cope with new situations and strangers.

*"The project group notes that it is important to be aware of the kind of residents they are dealing with and that non-participation does not necessarily have to do with a lack of interest but to the fact that it requires a mental surmounting for many residents to enter into a new context with strangers or the like*. *Perseverance in the contact is important here*.*"* (K, final evaluation) (Our own translation—edited by American Journal Experts).

The above-mentioned barriers indicate that it seems important to be aware of the challenges and needs of the target group to effectively recruit participants. For some groups and in some neighborhoods, a focus on language barriers might be crucial, whereas for other groups and in other neighborhoods, support and trust could be the main focus. The key is to identify all the needs of the different groups in the neighborhoods.

#### Geographical challenges

Recruitment challenges are also linked to geographical factors, especially in rural areas. One challenge is geographical delimitation; the number of residents in the project area may be too small and therefore may create difficulty in recruiting enough participants for the intervention (F). Geographical distance to the place of the interventions is also an identified barrier for participation, such as if the participants have to spend time and money to get there (F, H, I).

*"An important prerequisite for the communities is the local placement of the meeting points*. *Many feel like the resident who declares that the distance is crucial*. *A thing that might seem trivial for someone (3 kilometers and 20 Danish crowns) might be a considerable barrier to participate in social communities for others if they have to spend time and money to get to and from the activity*.*"* (H, final evaluation) (Our own translation—edited by American Journal Experts).

Further, the physical distance and the lack of a natural local meeting point are recruitment challenges, especially in rural areas, because it is difficult to contact the residents.

*"It has not been possible to reach the residents at the local area adequately*. *First of all*, *this might be due to the geographical isolation of the peninsula from the rest of the municipality*. *Generally speaking*, *the location of the local area 20 kilometers from the municipal health promotion and prevention interventions in health centers in the municipality mean that no one from the local area uses the health center services*.*”* (I, project description) (Our own translation—edited by American Journal Experts).

Thus, projects in rural areas may have greater challenges with regard to recruitment, and projects in urban areas may have greater potential for close physical contact with the residents. The local context inevitably affects the interventions and their effects. Because of differences in the neighborhoods’ characteristics, such as the number of residents or a rural or urban location, there may be differences in the way the project employees recruit residents and organize interventions.

### Positive experiences

Positive experiences were related to the municipalities’ use of knowledge about the target groups’ needs. Positive experiences with recruitment were divided into two themes: *relation and trust* and *adjustment in the recruitment strategy*.

#### Relation and trust

The analysis showed that trust and positive relations are an important part of the recruitment of marginalized groups because they make it easier for residents to seek out and participate in interventions (A, B, D, E, H, J, K).

*"It is estimated that an important part of the project employees’ work is to create relations to the residents in the neighborhood due to the effectiveness of the personal contact to involve new residents*.*"* (K, midterm evaluation) (Our own translation—edited by American Journal Experts).

One way to build trust and relationships with the residents is through a proactive approach. The proactive approach is often described as a good way to recruit participants, and the most effective proactive approach seems to be face-to-face contact, such as “knocking on the door” or recruitment through other interventions. The face-to-face approach may compensate for some of the challenges experienced in relation to the recruitment of the target group, such as by compensating for some language barriers. This was the case in one of the municipalities where a bilingual employee reached many of the ethnic minorities (E). The work of bilingual employees was an advantage in this neighborhood, where one-third of the residents were immigrants.

Another way of building trust and relationships with the residents was through a local project office or meeting point (D, E, H, J, K). The presence of the project employees in the neighborhood provided some of the same benefits as proactive recruitment because trust between the residents and project employees had time to grow.

*"The availability in the neighborhood*, *the knowledge of the project and a safe relationship with the employees become factors that all play a role in whether proactive recruitment for prevention interventions succeeds*.*"* (B, final evaluation) (Our own translation—edited by American Journal Experts).

Trust in the project and employees can also be spread by the residents as they begin to recruit each other. In this way, positive stories about the interventions are spread and create a feeling of confidence, which can benefit recruitment efforts.

*“*… *It is the communication*, *where you [the resident] come and say*, *"Are you coming*?*" That works*… *it is the personal contact*, *where you turn to others*, *which works*.*”* (J, final evaluation) (Our own translation—edited by American Journal Experts).

#### Adjustment of the recruitment strategy

Seven of twelve municipalities adjusted their recruitment approaches during the implementation process if they experienced challenges in recruiting the intended group (A, B, C, D, E, F, J). One municipality adjusted the recruitment strategy after its midterm evaluation showed that the interventions did not reach the most vulnerable residents (E). The recruitment strategy involved information through written material at the beginning of the project but was changed to a proactive approach with face-to-face contact. The project employees examined which residents they did not reach, partly through the help of residents who had already participated, and then began to seek out this group, which resulted in contact with more residents who were vulnerable.

*"A major effort has been made*, *especially within the last six months before the final evaluation*, *to get in touch with the group of residents who are vulnerable*, *mentally ill or lonely and therefore do not of their own initiative seek Healthy Zone [project name]*. *This work has been fruitful*, *and Healthy Zone has made contact with a group of these residents*…*"* (E, final evaluation) (Our own translation—edited by American Journal Experts).

This change had a positive effect and indicates that evaluation and adjustment of the recruitment strategy during the process is important and effective, but it is possible that a positive effect would have emerged earlier if a proactive approach had been present from the start.

In addition to adjustment, another municipality learned that the name of the intervention could negatively affect participation. By changing the name, more residents were recruited (B). It therefore seems that the election of a recruitment approach is not the only important component when trying to reach a marginalized group.

*“As Healthy Friendship [project name] started marketing lifestyle talks as body age measurements and moved the offer to one of the community houses*, *the interest increased a bit*. *According to the project employees’ own reflections*, *the demand increased because body age measurements are “more catchy and known from TV”*. *…By changing “the marketing strategy” and changing the name of the activity*, *Healthy Friendship experienced an increase in participants”*. (B, final evaluation) (Our own translation—edited by American Journal Experts).

These examples indicate that making adjustments to the recruitment approach seemed to be an effective strategy for municipalities to improve recruitment. Thus, it seems important that the adjustments are made on the basis of evaluations of the target group’s needs and not on the basis of limited resources. One municipality found that proactive phone calls were very demanding; they eventually made adjustments and let students make the calls because there were not enough employees. This did not have a positive effect, and the evaluation showed that fewer residents were recruited (F).

## Discussion

The study revealed both positive experiences and barriers when recruiting marginalized groups in deprived neighborhoods. The barriers included challenges with the target group and geographical difficulties, and both depended on the particular context. The challenges with the target group involved distrust, language barriers in neighborhoods with many different nationalities, and a lack of ability to cope with new situations in vulnerable groups. Distrust and a lack of coping abilities might cause residents to lack the courage and desire to participate in the health program, whereas the language barrier can make it difficult for residents to read the written material that provides information about the activities. Geographical difficulties involved rural areas due to the lack of a local meeting point and distance to the activities. Furthermore, participation was challenged due to issues of time and money required to access the activities. The positive experiences included maintaining a local presence and face-to-face contact, which can improve the relationships and trust between the project employees and the marginalized groups and thereby increase participation in interventions. Residents’ recruitment of each other also improved participation. Furthermore, adjustments to the recruitment strategy based on continuously identifying and recognizing the target groups’ needs while the health program was in progress seemed to be an effective way to improve participation. The analysis showed that adjustments succeeded if the project employees continuously identified which groups of residents they did not reach and then targeted the recruitment approach to these groups.

Other studies are in line with our findings of a positive experience based on a personal and proactive recruitment approach. A study by Macleod et al. (2013) examined recruitment approaches for overweight or obese post-partum women living in a deprived area in Tayside, Scotland, which partly might be comparable to the neighborhoods in Denmark. The results showed that the most effective method for recruiting was visiting community groups; accordingly, that project staff’s visibility in the area was an advantage [25]. Similar results were reported in a review by Koopmans et al. (2012), which indicated that more active and personal approaches yielded greater participation in prevention programs in General Practice than passive approaches did among individuals from deprived areas [12]. A randomized controlled trial by Heinrichs (2006) examined payment as a recruitment approach. Although payment is not included in FIN, the study by Heinrichs is interesting as the context is comparable to the Danish deprived neighborhoods. The study showed that paying residents for participation in an intervention concerning improvements to parenting skills was effective in increasing participation for at least one session. Once the participants experienced the benefit of the intervention, they seemed to become self-motivated to attend regularly, and payment was less pertinent. The study was more effective among families from deprived neighborhoods than among families from more affluent neighborhoods [26]. Therefore, the use of payment might be considered to improve recruitment among marginalized groups in deprived neighborhoods in Denmark.

International studies and researchers focusing on deprived neighborhoods and health emphasize the importance of a socio-ecological approach [27–29]. The socio-ecological approach involves the understanding that health is created in the interaction between citizens and the environment in which they live [28]. Trickett emphasizes problems with the use of decontextualized knowledge when conducting primary prevention projects and highlights that the focus should be not only on the intervention but also on the context in which the intervention is conducted [27]. Therefore, when choosing or planning a recruitment strategy, it is important to consider the context in which interventions should be implemented. Our analysis identified several examples of how the context influenced the choices made in relation to the recruitment strategy. One example was hiring a bilingual employee in a neighborhood where many of the residents were immigrants (E). Furthermore, it might be worth testing whether free transport in rural areas can increase participation due to the distance barriers for many residents (F, H, I).

In the documents, there was a lack of evaluation and follow-up concerning the target group. This might be explained by the lack of requirements and support from the Danish Health Authority in terms of documentation and evaluation of the interventions. This lack of requirements might be a reason for the general trend of municipalities not describing in detail the recruitment process and the adjustments they made throughout the process. International research suggests using an iterative approach when working with health interventions [28]. This approach involves continuous evaluations and adjustments of the interventions and recruitment approaches to reach the target group as well as to handle unexpected changes in the environment. The iterative approach requires knowledge about the socio-ecological condition, an ongoing evaluation and a systematic approach using theory, evidence and experience in the specific practice [28]. A systematic review by Bonevski et al. (2014) emphasized the importance of acknowledging the need for an extended time frame, higher resource needs and operation via community partnerships in an attempt to access hard-to-reach-groups [16]. Our study showed that limited resources, such as economic limitations, lack of time, and lack of experience and knowledge with systematic working in practice, might present challenges in relation to adjusting the recruitment approaches to the target group’s needs. We also observed that three municipalities that involved researchers or external evaluators were more systematic in their documentation and were the only ones that evaluated recruitment in relation to marginalized groups. The involvement of researchers throughout the entire process is also recommended [28].

Our study’s results are based on the fact that only 10 municipalities developed evaluations of recruitment, and only three evaluations provide a description of which residents were recruited to determine whether the residents belonged to one of the marginalized groups in the neighborhood. Some municipalities’ documents were more useful in the analysis than others because these municipalities had been more systematic and diligent in their description and evaluation of the recruitment of the target group. The defective documents made it difficult to determine whether the recruitment process had succeeded. Our method may not capture all of the useful experiences of FIN, and it could have been supplemented with interviews with project employees or residents, who may have different perspectives than those represented in the documents.

## Conclusion

This study provides important information on the process of recruiting marginalized groups in deprived neighborhoods for health interventions. Positive recruitment experiences involve the establishment of trust between the residents and the project employees. Trust can be achieved through the employees’ presence in the neighborhood, face-to-face contact and residents’ recruitment of each other. Furthermore, adjusting some of the interventions and the recruitment strategy increased participation. Further research is necessary to determine effective recruitment approaches to health interventions among marginalized groups. We recommend a systematic process of evaluation to examine which approaches are effective under which circumstances in relation to marginalized groups. Moreover, the collection of data during the recruitment process must include both qualitative methods that explore project employees' and residents' experiences and quantitative measures that assess participation development.

## References

[1] Marmot M, Review of social determinants and the health divide in the WHO European Region: final report. Copenhagen: World Health Organization, Regional Office for Europe; 2014.

[2] Diderichsen F, Andersen I, Manuel C, Andersen A-MN, Bach E, Baadsgaard M, m.flHealth Inequality—determinants and policies. Scand J Public Health. 1 11 2012;40(8 Suppl):12–105. 10.1177/1403494812457734 23147863

[3] van Lenthe FJ, Borrell LN, Costa G, Diez Roux AV, Kauppinen TM, Marinacci C, et al Neighbourhood unemployment and all cause mortality: a comparison of six countries. J Epidemiol Community Health. 1 3 2005;59(3):231–7. 15709084 10.1136/jech.2004.022574 PMC1733024

[4] Sundhedsstyrelsen. Center for forebyggelse [The Danish Health Authority. Center for Prevention]. Opslag af satspulje”Forebyggelsesindsatser i nærmiljøet” [Notice for special pool for the social area “Prevention efforts in the local community”]. 2010.

[5] Algren MH, Bak CK, Berg-Beckhoff G, Andersen PT. Health-Risk Behaviour in Deprived Neighbourhoods Compared with Non-Deprived Neighbourhoods: A Systematic Literature Review of Quantitative Observational Studies. PLOS ONE. 27 10 2015. 10.1371/journal.pone.0139297 PMC4624433 26506251

[6] Bak CK, Andersen PT, Dokkedal U. The association between social position and self-rated health in 10 deprived neighbourhoods. BMC Public Health. 2015;15(1):14. 25605136 10.1186/s12889-015-1377-2 PMC4308888

[7] Poortinga W, Dunstan FD, Fone DL. Neighbourhood deprivation and self-rated health: The role of perceptions of the neighbourhood and of housing problems. Health Place. 9 2008;14(3):562–75. 17997343 10.1016/j.healthplace.2007.10.003

[8] Pickett KE, Pearl M. Multilevel analyses of neighbourhood socioeconomic context and health outcomes: a critical review. J Epidemiol Community Health. 1 2 2001;55(2):111–22. 11154250 10.1136/jech.55.2.111 PMC1731829

[9] Kawachi I, Berkman LF. Neighborhoods and Health. Oxford University Press; 2003.

[10] Riva M, Gauvin L, Barnett TA. Toward the next generation of research into small area effects on health: a synthesis of multilevel investigations published since Jul. 1998. J Epidemiol Community Health. 10 2007;61(10):853–61. 17873220 10.1136/jech.2006.050740 PMC2652961

[11] Ellaway A, Benzeval M, Green M, Leyland A, Macintyre S. “Getting sicker quicker”: does living in a more deprived neighbourhood mean your health deteriorates faster? Health Place. Mar. 2012;18(2):132–7. 10.1016/j.healthplace.2011.08.005 PMC3391685 21873103

[12] Koopmans B, Nielen MM, Schellevis FG, Korevaar JC. Non-participation in population-based disease prevention programs in general practice. BMC Public Health. 2012;12(1):856. 23046688 10.1186/1471-2458-12-856 PMC3490995

[13] Goyder EC, Botha JL, McNally PG. Inequalities in access to diabetes care: evidence from a historical cohort study. Qual Health Care. 2000;9(2):85–9. 11067256 10.1136/qhc.9.2.85 PMC1743518

[14] Ouédraogo S, Dabakuyo-Yonli TS, Roussot A, Pornet C, Sarlin N, Lunaud P, et al European transnational ecological deprivation index and participation in population-based breast cancer screening programmes in France. Prev Med. 6 2014;63:103–8. 10.1016/j.ypmed.2013.12.007 24345603

[15] Bender AM, Kawachi I, Jørgensen T, Pisinger C. Neighborhood deprivation is strongly associated with participation in a population-based health check. PLOS ONE. 3 6 2015. 10.1371/journal.pone.0129819 PMC4454539 26039635

[16] Bonevski B, Randell M, Paul C, Chapman K, Twyman L, Bryant J, et al Reaching the hard-to-reach: a systematic review of strategies for improving health and medical research with socially disadvantaged groups. BMC Med Res Methodol. 2014;14:42 10.1186/1471-2288-14-42 24669751 PMC3974746

[17] Danmarks Statistik [Statistics Denmark]. Statistik for kommuner [Statistics for municipalities]. 2010. Available at: www.dst.dk. The included data were obtained from annual records of statistics for municipalities.

[18] Ministeriet for by, bolig og landdistrikter [Ministry of city, housing and rural district]. Regional- og Landdistriktspolitisk Redegørelse 2014. Regeringens redegørelse til Folketinget [Regional and rural district political statement 2014. The government’s statement to the national parliament of Denmark]. 2014.

[19] Danmarks Statistik [Statistics Denmark]. Indvandrere og efterkommere [Immigrants and descendants]. 2016. Available at: https://www.dst.dk/da/Statistik/emner/indvandrere-og-efterkommere/indvandrere-og-efterkommere?tab=dok

[20] Danmarks Statistik [Statistics Denmark]. Statistik for indkomst [Statistics for income]. 2008. Available at: www.dst.dk.

[21] Beskæftigelsesministeriet [Ministry of Employment]. Betingelser for at få kontanthjælp [Conditions for obtaining social assistance]. 2016. Available at: http://bm.dk/da/Beskaeftigelsesomraadet/Ydelser/Kontanthjaelp/Betingelser%20for%20at%20faa%20kontanthjaelp.aspx

[22] Saunders RP. Developing a Process-Evaluation Plan for Assessing Health Promotion Program Implementation: A How-To Guide. Health Promot Pract. 1 4 2005;6(2):134–47. 15855283 10.1177/1524839904273387

[23] Hsieh H-F. Three Approaches to Qualitative Content Analysis. Qual Health Res. 1 11 2005;15(9):1277–88. 16204405 10.1177/1049732305276687

[24] Coffey A, Atkinson P. Making Sense of Qualitative Data: Complementary Research Strategies. 1 edition Thousand Oaks: SAGE Publications, Inc; 1996.

[25] Macleod M, Craigie AM, Barton KL, Treweek S, Anderson AS. Recruiting and retaining postpartum women from areas of social disadvantage in a weight-loss trial—an assessment of strategies employed in the WeighWell feasibility study. Matern Child Nutr. 7 2013;9(3):322–31. 10.1111/j.1740-8709.2011.00393.x 22284216 PMC6860823

[26] Heinrichs N. The Effects of Two Different Incentives on Recruitment Rates of Families into a Prevention Program. J Prim Prev. 7 2006;27(4):345–65. 16802074 10.1007/s10935-006-0038-8

[27] Trickett EJ. Toward a Framework for Defining and Resolving Ethical issues in the Protection of Communities Involved in Primary Prevention Projects. Ethics Behav. 12 1998;8(4):321–37. 11660541 10.1207/s15327019eb0804_5

[28] Edwards N, Mill J, Kothari AR. Multiple Intervention Research Programs in Community Health. CJNR Can J Nurs Res. 2004;36(1):40–54. 15133918

[29] Wall M, Hayes R, Moore D, Petticrew M, Clow A, Schmidt E, et al Evaluation of community level interventions to address social and structural determinants of health: a cluster randomised controlled trial. BMC Public Health. 2009;9(1):207. 19558712 10.1186/1471-2458-9-207 PMC2713231

